# The Association Between Female Smoking and Childhood Asthma Prevalence–A Study Based on Aggregative Data

**DOI:** 10.3389/fpubh.2018.00295

**Published:** 2018-10-17

**Authors:** Vishal Midya, Shekhar Pal, Ankita Sinharoy, Jishu K. Das, Harish Rao, Mutasim Abu-Hasan, Pritish Mondal

**Affiliations:** ^1^Division of Biostatistics and Bioinformatics, College of Medicine, Pennsylvania State University, Hershey, PA, United States; ^2^Calcutta School of Tropical Medicine, Kolkata, India; ^3^Penn State Milton S. Hershey Medical Center, Hershey, PA, United States; ^4^Division of Medical Oncology, The Ohio State University, Columbus, OH, United States; ^5^Department of Pediatrics, University of Florida, Gainesville, FL, United States

**Keywords:** childhood asthma prevalence, socioenvironmental predictors of asthma, global asthma network, pediatric asthma, female smoking, human developmental index

## Abstract

**Aims:** Socioeconomic and environmental factors influence childhood asthma prevalence across the world. In-depth epidemiological research is necessary to determine the association between asthma prevalence and socio-environmental conditions, and to develop public health strategies to protect the asthmatic children against the environmental precipitators. Our research was based on aggregative data and sought to compare the asthma prevalence between children of two different age-groups across the world and to identify the association among the key socio-environmental conditions with increased childhood asthma prevalence.

**Method:** We included forty countries with available data on various socio-environmental conditions (2014–2015). Childhood asthma prevalence of two different age groups (6–7 and 13–14 years) were obtained from global asthma report 2014. Because of significant diversities, the selected countries were divided into two groups based on human developmental index (HDI), a well-recognized parameter to estimate the overall socioeconomic status of a country. Robust linear regression was conducted using childhood asthma prevalence as the dependent variable and female smoking prevalence, tertiary school enrollment (TSE), PM_10_ (particulate matter ≤10 μm in diameter) and gross domestic product (GDP) as predictors.

**Results:** Asthma prevalence was not different between two age groups. Among all predictors, only female smoking prevalence (reflecting maternal smoking) was associated with asthma prevalence in the countries with lower socio-economic conditions (HDI), but not in the higher HDI group. The results were unchanged even after randomization.

**Conclusions:** Childhood asthma prevalence did not change significantly with age. Female smoking may have a positive correlation with childhood asthma prevalence in lower HDI countries.

## Introduction

Asthma is one of the most common noncommunicable diseases worldwide and has long been associated with various socioeconomic and environmental conditions. Weitzman et al. acknowledged the impact of socioenvironmental conditions on the outcome of childhood asthma in the United States as early as 1990 (1). Further, a previous study conducted by our team has demonstrated that asthma-related mortality was higher in the developing countries compared to developed ones, with lack of resources and poverty were likely to be the key contributing factors(2). However, very few global epidemiological studies have been conducted in the recent years to delineate further the impact of socio-environmental conditions on the prevalence of childhood asthma. Leading asthma researchers had previously surmised that the outcome of asthma primarily depends on IgE mediated eosinophilic inflammation or atopy (3). However, the International Study of Asthma and Allergies in Childhood (ISAAC) phase 3 study concluded that the increased childhood asthma prevalence observed in recent years, especially in developing countries, may be influenced by other environmental factors in addition to atopy^1^ (4). These findings constituted a conceptual shift in the asthma epidemiology and emphasized the necessity of further investigation to determine other underlying precipitators of childhood asthma^1^. ISAAC was terminated in 2012, and since then Global Asthma Network (GAN) has been responsible for carrying forward its mission^2^.

Childhood asthma prevalence varies significantly not only between affluent and poor countries^2^, but also differs in immigrants when compared to the inhabitants of their country of inheritance. For example, the ISAAC phase 3 trial indicated that asthma symptoms were highest among the Chinese-Canadian adolescents (born and raised in Canada), followed by the children who were born in China and later immigrated to Canada (>7 years back), while the symptoms were lowest in the Chinese adolescents who had never migrated (5). One might then infer that the Canadian socioenvironmental conditions could be accountable for the increased asthma prevalence among Canadians of Chinese origin since the asthma prevalence in Canada is higher compared to China.

Parental asthma education helps to promote greater awareness which can facilitate a favorable outcome of childhood asthma (6). Most of the developed countries with a higher gross domestic product (GDP) can offer better health care to their citizen. In contrast, children from the poorest parts of the society are worst sufferers of any chronic diseases due to lack of access to the healthcare and poor living conditions. Wisow et al. demonstrated that the rate of asthma-related admission at a Maryland hospital was higher among the African-Americans and that was largely contributed by poverty (7). Although asthma-related mortality is significantly higher in poorer countries than that of the developed ones, asthma prevalence does not follow a similar trend (2, 8). ISAAC cited a higher prevalence of wheeze among the adolescents (13–14 years) of affluent countries (9).

Maternal smoking and even third-hand smoking is known to affect the frequency and severity of childhood asthma (10, 11). Kabesch et al. demonstrated that second-hand smoke exposure due to maternal smoking often led to wheezing in younger children (0–6 years). However, that influence tends to decline in older children suggesting that the impact of socioeconomic conditions may differ among different age groups (younger children vs. adolescents). Uncontrolled air pollution is another important environmental factor causing increased asthma prevalence. A meta-analysis of 36 studies by Weinmayr et al. has demonstrated a strong association between higher PM_10_ (particulate matter ≤10 μm in diameter) and increased asthma prevalence in children (12). Association analysis between air pollution and adult asthma outcome by Maestrelli et al. suggested that PM_10_ concentration had influenced the asthma-related morbidity but not PM_2.5_ (13).

With this background, we hypothesized that in addition to atopy and racial heterogeneity, various socio-economic and environmental conditions might contribute to the disparity in asthma prevalence throughout the world. Thus, our study was aimed at determining the influences of four specific socioeconomic and environmental conditions on childhood asthma prevalence in different countries: female smoking prevalence, tertiary school enrollment, PM_10_, and GDP. To test this hypothesis, we developed a unique approach to analyzing and correlating international aggregative datasets obtained by various globally recognized organizations. We included group of 6–7 years old and 13–14 years old children following the categorization by GAN^3^.

## Methods

### Sample selection

We selected 60 countries for this cross-sectional study using a random selection tool^4^. Forty out of those had most of the required data points available from 2014 to 2015, thus were considered for data analysis (Supplemental Figure 1).

### Childhood asthma prevalence

Following the GAN categorization, prevalence data of two age-groups were included^2^: 6–7 years old and 13–14 years old.

### Parameters of socioeconomic and environmental conditions

GDP (per capita) and tertiary school enrollment (TSE) were included as the socio-economic conditions, while female smoking prevalence (age>15 years) and PM_10_ (μg/m^3^) were considered as environmental risk factors. We have utilized various globally recognized organizations resources for data collection (Supplemental Table 1)^5^^,^^6^^,^^7^^,^^8^^,^^9^^,^ ^10^

### Sample size estimation

The correlation analysis between GDP and asthma prevalence (6–7 years) was used as a model for the sample size estimation using G^*^power^11^. The correlation coefficient between those two variables was 0.47. The estimated required sample size was 32 [input criteria:0.8 of power(1-β), α = 0.05, two tails].

### Stratification according to socioeconomic conditions (HDI)

Since we selected countries with extremely diverse conditions from six continents, stratifying them into groups according to their socioenvironmental status was necessary. Hence, a standard parameter was required to achieve that categorization. United Nations Development Program has accepted HDI as the ultimate parameter to assess the overall socioeconomic well being of a country, instead of focusing on isolated GDP^12^. HDI was created assimilating multiple parameters including life expectancy, education index, and GDP. Thereby, we utilized HDI to stratify selected countries into higher and lower group through the median (Supplemental Figure 1).

### Statistical analyses

We used R (3.5.1) and IBM SPSS (version 24) for data analysis and Mann Whitney *U*-test for two-sample comparisons. Robust linear regression was used for both the HDI groups considering non-normality of data, with asthma prevalence as the dependent variable; while female smoking prevalence, TSE, PM_10_, and GDP were included as the predictors. For reproducibility, 16 out of 20 countries were randomly selected 1,000 times by SRSWOR function, and robust regressions were re-run to estimate the proportion of times each predictor was significant in each HDI groups. Two-tailed *p*-values of <0.05 were considered statistically significant.

## Results

### Lower HDI countries

Asthma prevalence in 6–7 years old children [as expressed in median (IQR)] 5.00 (6.20) was not significantly different from that of 13–14 years old children 10.80 (7.90) (*p* = 0.17 derived from Mann Whitney test). GDP and female smoking prevalence had strong association [correlation coefficient (r) = 0.66, *p* = 0.001] (Supplemental Figure 2). However, TSE did not have a significant correlation with GDP or female smoking.

### Higher HDI countries

Asthma prevalence in 6–7 years old children 11.00 (12.70) was not significantly different from that of older children 14.10 (11.20) (*p* = 0.51 derived from Mann Whitney test). GDP, TSE and female smoking prevalence did not have a significant correlation among themselves (Supplemental Figure 3).

### Comparative analyses (mann whitney *U*-Test- Table 1)

Low HDI countries compared to high HDI, had significantly lower asthma prevalence rates in both age groups (*p* = 0.043 and *p* = 0.023, respectively for younger and older children). However, Asthma prevalence was not significantly different between younger and older children (*p*-values of 0.21 and 0.75 for low and high HDI groups, respectively). Expectedly, countries with lower HDI had worse socio-economic conditions like GDP and TSE in contrast to higher HDI group (*p* < 0.001 for both the variables). Lower HDI countries also had a worse level of air pollution (PM_10_) and decreased female smoking prevalence in comparison to higher HDI countries (*p* < 0.001 and *p* = 0.005, respectively).

**Table 1:** Mann Whitney *U*-test demonstrating the significant difference between asthma prevalence and socio-environmental predictors between lower and higher HDI countries.

	**Lower HDI countries**			**Higher HDI countries**			***p*-value**
	**Mean ± SD**	**Median**	IQR^*^	**Mean ± SD**	**Median**	**IQR**	
Asthma Prevalence (6–7 years)	8.76 ± 9.40	5.00	6.20	14.83 ± 7.77	11.00	12.70	0.003
Asthma Prevalence (13–14 years)	10.29 ± 7.55	10.80	7.90	15.78 ± 8.63	14.10	11.20	0.014
GDP ($/capita)	7201 ± 9714	4529	5354	29833 ± 17739	21930	27485	<0.001
Female smoking prevalence	5.78 ± 5.19	3.60	6.95	13.49 ± 8.26	13.70	16.55	0.005
TSE (% of population)	38.79 ± 16.83	40.48	27.07	66.48 ± 18.28	63.38	31.59	<0.001
PM10 (μg/m^3^)	82.92 ± 82.70	48.00	46.40	32.45 ± 21.54	27.30	12.50	<0.001

*P-values (two-tailed significance level) are derived from Mann Whitney U test comparing lower vs. higher HDI parameters*.

* *Interquartile Range*.

*$ = USD*.

### Robust linear regression

#### Low HDI countries

Female smoking prevalence was the only predictor variable that was statistically significant for asthma prevalence in both age groups while controlling for PM10, GDP, and TSE (*p* < 0.01 and *p* = 0.01 for the younger and older age groups, respectively; Table 2).

**Table 2:** Regression coefficients from robust linear regression^#^ based on 20 countries in each of the lower and higher HDI groups.

**Regression parameters**	**Asthma prevalence 6 to 7 years (Lower HDI)**		**Asthma prevalence 13 to 14 years (Lower HDI)**		**Asthma prevalence 6 to 7 years (Higher HDI)**		**Asthma prevalence 13 to 14 years (Higher HDI)**	
	***t*-statistics (df = 14)**	***p*-value**	***t-*statistics (df = 15)**	***p-*value**	***t-*statistics (df = 13)**	***p*-value**	***t-*statistics (df = 14)**	***p*-value**
Female Smoking	3.61	0.003^**^	2.81	0.01^*^	−1.46	0.17	−0.19	0.85
GDP	0.04	0.97	0.85	0.41	1.48	0.16	1.99	0.07
PM10	−0.27	0.79	−0.96	0.35	−0.62	0.54	0.75	0.47
TSE	−0.9	0.38	−1.99	0.06	−0.12	0.9	0.05	0.96

*The analysis demonstrated that for lower HDI countries, female smoking prevalence was significant predictors of asthma prevalence (in both age groups). Likewise, for higher HDI countries, none of the independent variables were significant*.

# M-estimation was employed along with Hampel Psi function and for scale estimation re-scaled mean absolute deviation (MAD) of residuals was used; p-value:

* < 0.05;

** *< 0.01*.

### High HDI countries

None of the predictors variable including female smoking prevalence was statistically significant (Table 2).

### Random sampling within strata (HDI groups)

In low HDI countries, female smoking prevalence was a significant predictor for asthma prevalence (in both age groups) in more than 80% simulations (Supplemental Figure 4). Likewise, for high HDI countries, none of the independent variables were significant in most of the simulations.

### Other methods

Regression analyses results were reproduced using linear mixed models (Supplemental Table 2) showing high conformity.

## Discussion

**“**Growing out of childhood asthma in adolescence” is a well-known concept and the hypothesized mechanism is the reduction of asthma prevalence due to the immunological changes induced by sex hormones at puberty (14). However, according to R Zannolli, this concept was not supported by scientific evidence (15). In contrast, recent studies suggested that asthma symptoms may worsen in girls during puberty (16). We did not find a difference in asthma prevalence between younger and teenage children. However, the asthma prevalence was significantly lower in countries with low HDI compared to high HDI. The rationale behind this observation was not well understood. An inefficient data collection system and inadequate diagnostic facilities in the underdeveloped countries might be attributable for an underestimation of prevalence (2).

Our study indicates that the female smoking prevalence (reflecting maternal smoking) was reduced in low HDI countries compared to higher HDI, which could have been influenced by sociocultural practices, stigma, and lack of financial freedom among women in under-developed countries (2). More importantly, the regression analyses showed that female smoking prevalence, was the only predictor of childhood asthma prevalence in countries with lower socioeconomic conditions. It was surprising to find the lack of association between asthma prevalence and any of the socio-environmental factors in higher HDI countries, despite an increased asthma prevalence and higher values of the predictors (GDP, TSE, and female smoking prevalence). A likely explanation could be that the increased asthma prevalence in high HDI countries was a result of multi-factorial contribution and none of the predictors individually was the single most important defining factor. Nonetheless, this result came out of a study based on aggregative data and could not oppose the impact of maternal smoking in the developed countries.

Female smoking, especially smoking during pregnancy, has a deleterious effect on the offspring, as it can induce epigenetic changes resulting in the increased risk of asthma and persistent wheezing in children (17, 18). Further, Tager et al. conducted a prospective study and described the progressive decline in lung functions in children who were persistently exposed to the maternal smoking as they grow older (19). Thus, based on the observations from several prospective studies, GAN recommended strict implementation of policies to reduce tobacco smoking and to avoid second-hand smoke exposure in children at both government and national organizational level to achieve the goal of reduced asthma prevalence by 50% by 2025^2^.

Limitations of this study include lack of applicability to any specific region since our research was based on aggregative data. Thus, a causal relationship could not be derived from such data, and it would require a prospective study. Also, sex-specific asthma prevalence analysis could not be done due to lack of available data. Reproducibility of regression results after 1,000 times randomization was a strength of this study and indicated that a similar outcome should be expected from future prospective studies.

In summary, this study enabled us to recognize that among various socioeconomic and environmental factors studied, female smoking prevalence possibly had the strongest association with increased asthma prevalence in countries with lower socio-economic conditions. Our result thus supports the recommendation of GAN report 2014, that national strategies to prevent secondhand smoke exposure in general and maternal smoking, in particular, would be helpful to achieve reduced global childhood asthma prevalence by 2025^2^.

## Conclusion

Asthma prevalence in children was not significantly different between younger children and adolescents. Female smoking was found to be one of the major socio-environmental predictors of increased asthma prevalence in children of different age groups from the countries with lower socio-economic conditions.

## Author contributions

PM was the principal investigator and was primarily responsible for manuscript writing. VM helped with concept, design of the study along-with PM. VM, PM and AS performed statistical analysis. PM, SP, JD and AS has was responsible for data collection. SP helped with study design and concept. HR, JD and MA-H helped to contribute with the clinical aspects along with PM. All the authors including PM, VM, SP, HR, JD and MA-H helped the final draft of the manuscript and approved.

### Conflict of interest statement

The authors declare that the research was conducted in the absence of any commercial or financial relationships that could be construed as a potential conflict of interest.

## Footnotes

^1^http://isaac.auckland.ac.nz/

^2^http://www.globalasthmanetwork.org/news/GAR2014.php [Internet].

^3^http://www.globalasthmanetwork.org/news/GAR2014.php

^4^www.randomlists.com/random-country

^5^https://www.cia.gov/library/publications/the-world-factbook/rankorder/2004rank.html

^6^http://databank.worldbank.org/data/reports.aspx?source=2&series=SE.TER.ENRR&country=# [Internet].

^7^National Accounts Main Aggregates Database for GDP. Available from: https://unstats.un.org/unsd/snaama/selbasicFast.asp

^8^http://apps.who.int/gho/data/node.main.65

^9^The tobacco Atlas. Available from: https://tobaccoatlas.org/topic/prevalence/

^10^http://aqicn.org/faq/2015-05-16/world-health-organization-2014-air-pollution-ranking/. [Internet].

^11^http://www.gpower.hhu.de/

^12^United Nations Development Programme and Human Development Index. Available from: http://hdr.undp.org/en/content/human-development-index-hdi

## Supplementary material

Supplemental Figure 1 Consort diagram delineating the outline of the study design.

Supplemental Figure 2 Scatter diagrams demonstrating significant associations between childhood asthma prevalence in two different age groups (6–7 years and 13–14 years of age) and female smoking prevalence in countries with lower socio-economic conditions (lower HDI; with *p* < 0.01 in both age groups, and *r* = 0.79 and *r* = 0.70, respectively).

Supplemental Figure 3 Scatter diagrams demonstrating lack of associations between childhood asthma prevalence in two different age groups and female smoking prevalence in countries with higher socioeconomic conditions (higher HDI; *p* > 0.05 for both age groups).

Supplemental Figure 4 Thousand times randomization using the SRSWOR function in R software demonstrating that for low HDI countries, female smoking prevalence was significant predictors for asthma prevalence (in both age groups) in more than 80% simulations. Likewise, for high HDI countries, none of the independent variables were significant in most of the simulations.
