# Supplementary figures and images for: The Association Between Female Smoking and Childhood Asthma Prevalence–A Study Based on Aggregative Data
Source: Front Public Health. 2018 Oct 17;6:295. doi: 10.3389/fpubh.2018.00295 (PMC6199460; doi:10.3389/fpubh.2018.00295)

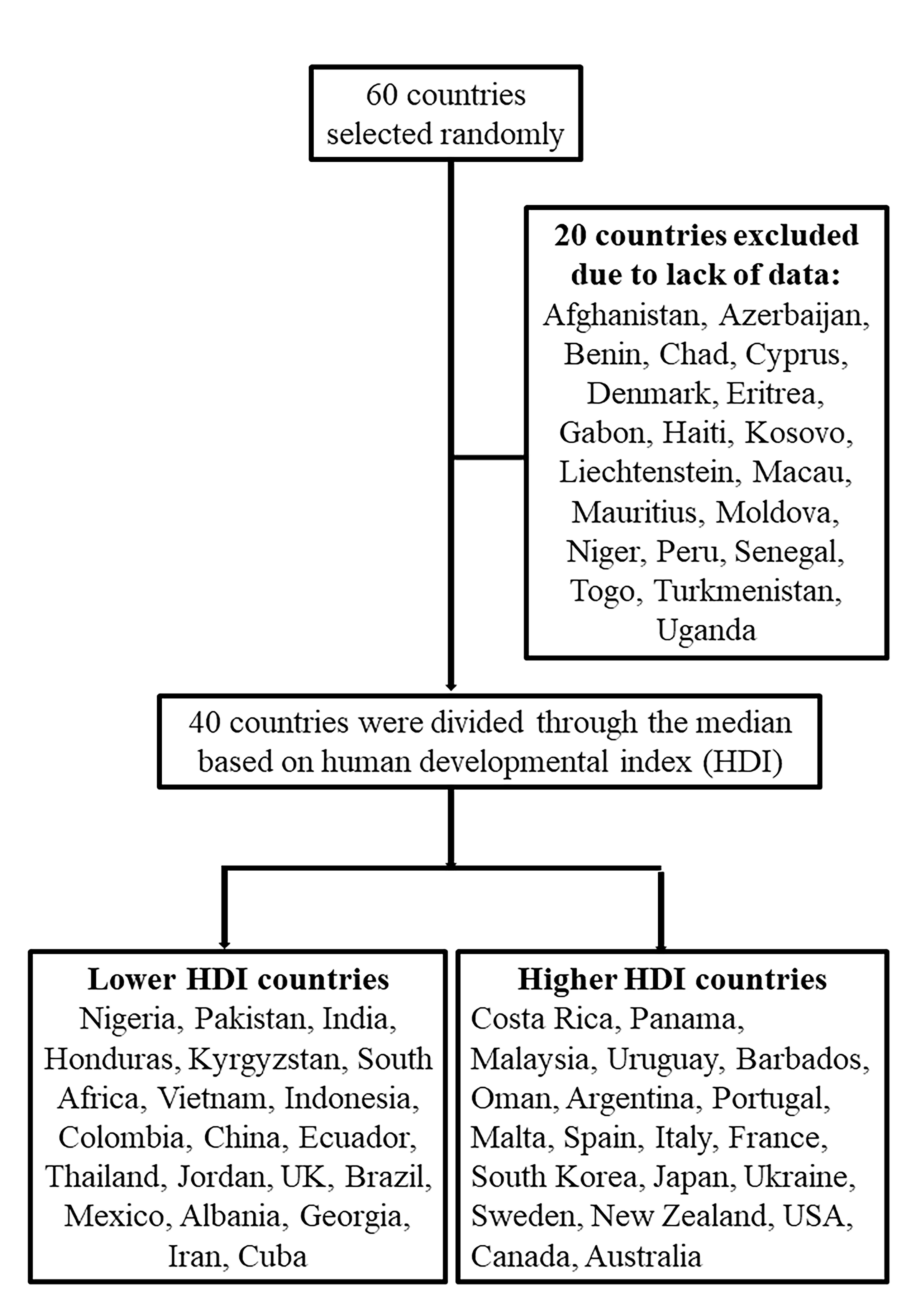

Supplement: Supplemental Figure 1 — Consort diagram delineating the outline of the study design. [file Image_1.TIF]

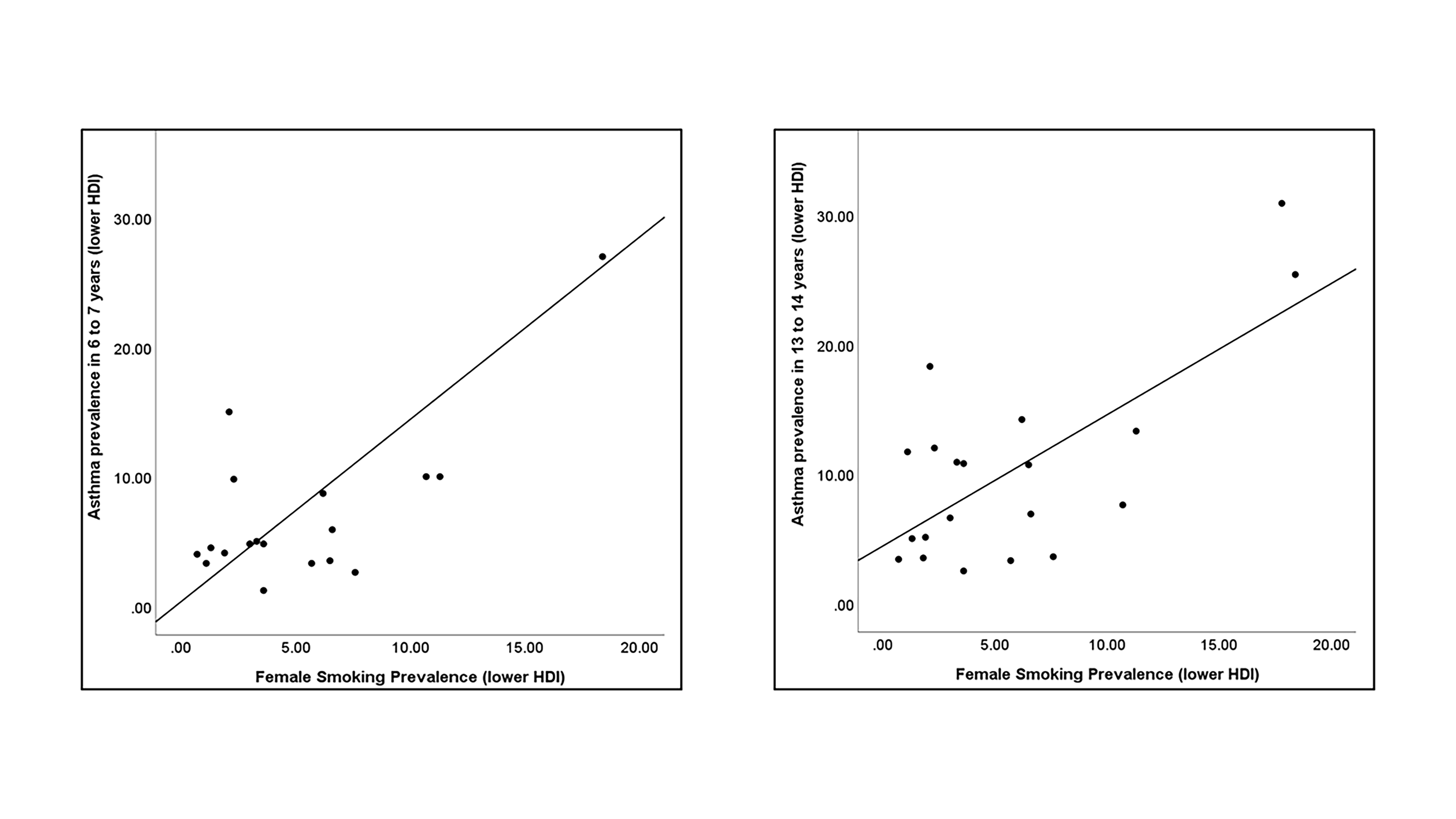

Supplement: Supplemental Figure 2 — Scatter diagrams demonstrating significant associations between childhood asthma prevalence in two different age groups (6–7 years and 13–14 years of age) and female smoking prevalence in countries with lower socio-economic conditions (lower HDI; with p < 0.01 in both age groups, and r = 0.79 and r = 0.70, respectively). [file Image_2.TIF]

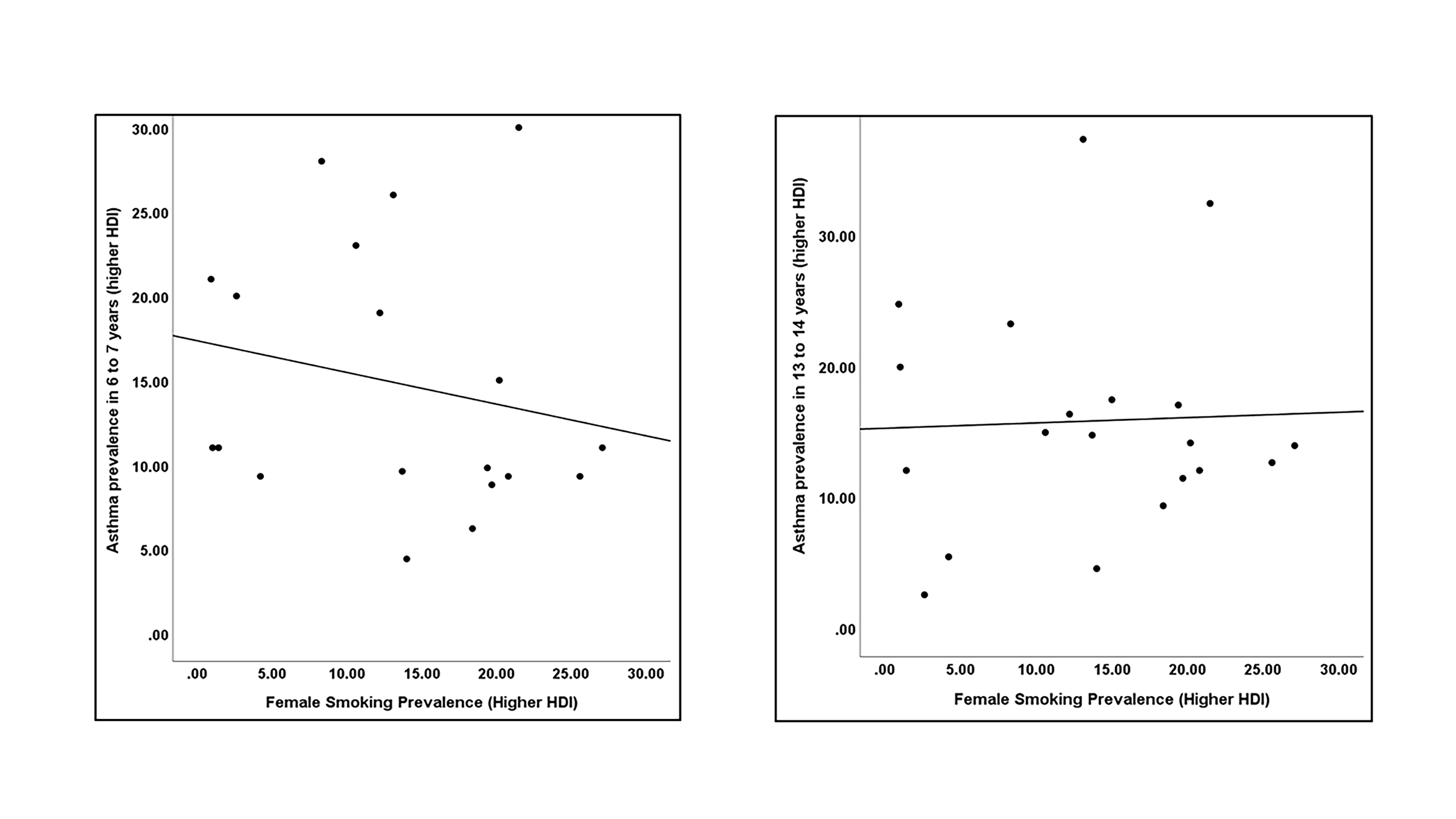

Supplement: Supplemental Figure 3 — Scatter diagrams demonstrating lack of associations between childhood asthma prevalence in two different age groups and female smoking prevalence in countries with higher socioeconomic conditions (higher HDI; p > 0.05 for both age groups). [file Image_3.TIF]
