# Supplementary material for: The Association Between Female Smoking and Childhood Asthma Prevalence–A Study Based on Aggregative Data
Source: Front Public Health. 2018 Oct 17;6:295. doi: 10.3389/fpubh.2018.00295 (PMC6199460; doi:10.3389/fpubh.2018.00295)
Supplement: Supplementary file 5 [file Table_1.DOCX]

Supplemental Table 1: Country-wise distribution of asthma prevalence in children of two different age-groups and their socio-environmental predictors (lower HDI countries are in clear rows, while higher HDI countries are shaded in gray)

|  | **HDI** | **Asthma 6to7 years (% of population)** | **Asthma 13to14 years (% of population)** | **GDP ($/ capita)** | **Female Smoking (% of population)** | **TSE (% of population)** | **PM10**  **(µg/m^3^)** |
| --- | --- | --- | --- | --- | --- | --- | --- |
| Nigeria | 0.53 | 3.30 | 11.70 | 3222 | 1.10 | 10.07 | 201.99 |
| Pakistan | 0.55 | 4.80 | 6.60 | 1342 | 3.00 | 10.34 | 364.80 |
| India | 0.62 | 4.10 | 5.10 | 1576 | 1.90 | 25.54 | 102.10 |
| Honduras | 0.63 | 15.00 | 18.30 | 2243 | 2.10 | 19.73 | 58.00 |
| Kyrgyzstan | 0.66 | 1.20 | 2.50 | 1293 | 3.60 | 46.27 | 35.00 |
| South Africa | 0.67 | 3.50 | 10.70 | 6433 | 6.50 | 19.78 | 53.40 |
| Vietnam | 0.68 | 4.50 | 5.00 | 2012 | 1.30 | 30.43 | 62.00 |
| Indonesia | 0.69 | 4.80 | 10.80 | 3492 | 3.60 | 29.56 | 48.00 |
| Colombia | 0.73 | 8.70 | 14.20 | 7913 | 6.20 | 53.28 | 41.20 |
| China | 0.74 | n/a | 3.50 | 7578 | 1.80 | 41.28 | 88.00 |
| Ecuador | 0.74 | 5.00 | 10.90 | 6432 | 3.30 | 40.48 | 36.30 |
| Thailand | 0.74 | 9.80 | 12.00 | 5942 | 2.30 | 50.17 | 41.40 |
| Jordan | 0.74 | 10.00 | 7.60 | 4067 | 10.70 | 44.87 | 128.00 |
| UK | 0.74 | 27.00 | 25.40 | 46494 | 18.40 | 57.30 | 36.80 |
| Brazil | 0.75 | 10.00 | 13.30 | 12027 | 11.30 | 49.28 | 33.78 |
| Mexico | 0.76 | 5.90 | 6.90 | 10581 | 6.60 | 30.23 | 61.80 |
| Albania | 0.76 | 2.60 | 3.60 | 4529 | 7.60 | 70.35 | 31.62 |
| Georgia | 0.77 | 3.30 | 3.30 | 4135 | 5.70 | 40.62 | 41.50 |
| Iran | 0.77 | 4.00 | 3.40 | 5662 | 0.70 | 65.62 | 177.30 |
| Cuba | 0.77 | 39.00 | 30.90 | 7050 | 17.80 | 40.54 | 15.49 |
| Costa Rica | 0.78 | 28.00 | 23.20 | 10647 | 8.30 | 53.04 | 32.80 |
| Panama | 0.79 | 20.00 | 2.50 | 12594 | 2.60 | 44.52 | 31.10 |
| Malaysia | 0.79 | 11.00 | 12.00 | 11184 | 1.40 | 36.87 | 27.00 |
| Uruguay | 0.79 | 9.80 | 17.00 | 16738 | 19.40 | 60.65 | 27.00 |
| Barbados | 0.80 | 21.00 | 24.70 | 15574 | 0.90 | 65.43 | 95.00 |
| Oman | 0.80 | 11.00 | 19.90 | 20458 | 1.00 | 35.31 | 82.00 |
| Argentina | 0.83 | 6.20 | 9.30 | 13193 | 18.40 | 82.92 | 30.00 |
| Portugal | 0.84 | 9.60 | 14.70 | 21930 | 13.70 | 65.15 | 27.30 |
| Malta | 0.86 | 15.00 | 14.10 | 26358 | 20.20 | 44.69 | 28.31 |
| Spain | 0.88 | 11.00 | 13.90 | 29597 | 27.10 | 88.88 | 22.80 |
| Italy | 0.89 | 8.80 | 11.40 | 36112 | 19.70 | 63.38 | 32.00 |
| France | 0.90 | 9.30 | 12.60 | 42924 | 25.60 | 64.10 | 24.20 |
| South Korea | 0.90 | 9.30 | 5.40 | 28011 | 4.20 | 93.42 | 51.10 |
| Japan | 0.90 | 23.00 | 14.90 | 37833 | 10.60 | 62.93 | 20.20 |
| Ukraine | 0.91 | 4.40 | 4.50 | 2974 | 14.00 | 83.42 | 19.60 |
| Sweden | 0.91 | 9.30 | 12.00 | 59221 | 20.80 | 62.18 | 17.30 |
| New Zealand | 0.91 | 30.00 | 32.40 | 44004 | 21.50 | 77.98 | 15.40 |
| USA | 0.92 | n/a | 17.40 | 54852 | 15.00 | 88.00 | n/a |
| Canada | 0.92 | 19.00 | 16.30 | 50355 | 12.20 | n/a | 20.70 |
| Australia | 0.94 | 26.00 | 37.30 | 62093 | 13.10 | 90.31 | 12.70 |

n/a= not applicable
