# Supplementary material for: The Association Between Female Smoking and Childhood Asthma Prevalence–A Study Based on Aggregative Data
Source: Front Public Health. 2018 Oct 17;6:295. doi: 10.3389/fpubh.2018.00295 (PMC6199460; doi:10.3389/fpubh.2018.00295)
Supplement: Supplementary file 6 [file Table_2.DOCX]

Supplemental Table 2: Regression coefficients from the linear mixed model reproduced the results of robust linear regression. The analysis demonstrated that for low HDI countries, female smoking prevalence was significant predictors for asthma prevalence (in both age groups). Likewise, for high HDI countries, none of the independent variables were significant.

|  | Asthma Prevalence 6 to 7 years  (Lower HDI) | | Asthma Prevalence 13 to 14 years (Lower HDI) | | Asthma Prevalence 6 to 7 years  (Higher HDI) | | Asthma Prevalence 13 to 14 years (Higher HDI) | |
| --- | --- | --- | --- | --- | --- | --- | --- | --- |
| Regression Parameters | t-statistics (df = 14) | p-value | t-statistics (df = 15) | p-value | t-statistics (df = 13) | p-value | t-statistics (df = 14) | p-value |
| Female Smoking | 3.61 | 0.002** | 2.81 | 0.01* | -1.46 | 0.17 | -0.19 | 0.85 |
| GDP | 0.04 | 0.97 | 0.85 | 0.41 | 1.48 | 0.16 | 1.99 | 0.07 |
| PM10 | -0.27 | 0.79 | -0.96 | 0.35 | -0.62 | 0.54 | 0.75 | 0.47 |
| TSE | -0.90 | 0.38 | -1.99 | 0.07 | -0.12 | 0.90 | 0.05 | 0.96 |

^#^ Random intercept was considered for each country; REML method was used for estimation; p-value: * < 0.05 ; ** < 0.01
